# Development of a PMMA phantom as a practical alternative for quality control of gamma knife® dosimetry

**DOI:** 10.1186/s13014-018-1117-8

**Published:** 2018-09-14

**Authors:** Jae Pil Chung, Young Min Seong, Tae Yeon Kim, Yona Choi, Tae Hoon Kim, Hyun Joon Choi, Chul Hee Min, Hamza Benmakhlouf, Kook Jin Chun, Hyun-Tai Chung

**Affiliations:** 1 Center for Ionizing Radiation, Division of Metrology for Quality of Life, Korea Research Institute of Standards and Science, 267 Gajeong-ro, Yuseong-gu, Daejon, 34311 Korea; 2 Department of Accelerator Science, Korea University Sejong Campus, 2511 Sejong-ro, Sejong, 30019 Korea; 3 Department of Nuclear Engineering, Hanyang University College of Engineering, Seoul, 04763 Korea; 4 Department of Radiation Convergence Engineering, Yonsei University, 1 Yeonsedae-gil, Heungeop-myeon, Wonju, 26493 Korea; 5 Department of Medical Radiation Physics and Nuclear Medicine, Karolinska University Hospital, SE-17176 Stockholm, Sweden; 6 Department of Neurosurgery, Seoul National University College of Medicine, 101 Daehak-ro Jongno-gu, Seoul, 03080 Korea

**Keywords:** Gamma knife, PMMA phantom, Quality control, Absorbed dose rate to water, Dose distribution, Penumbra, Scattered photon contribution

## Abstract

**Background:**

To measure the absorbed dose rate to water and penumbra of a Gamma Knife® (GK) using a polymethyl metacrylate (PMMA) phantom.

**Methods:**

A multi-purpose PMMA phantom was developed to measure the absorbed dose rate to water and the dose distribution of a GK. The phantom consists of a hemispherical outer phantom, one exchangeable cylindrical chamber-hosting inner phantom, and two film-hosting inner phantoms. The radius of the phantom was determined considering the electron density of the PMMA such that it corresponds to 8 g/cm^2^ water depth, which is the reference depth of the absorbed dose measurement of GK. The absorbed dose rate to water was measured with a PTW TN31010 chamber, and the dose distributions were measured with radiochromic films at the calibration center of a patient positioning system of a GK Perfexion. A spherical water-filled phantom with the same water equivalent depth was constructed as a reference phantom. The dose rate to water and dose distributions at the center of a circular field delimited by a 16-mm collimator were measured with the PMMA phantom at six GK Perfexion sites.

**Results:**

The radius of the PMMA phantom was determined to be 6.93 cm, corresponding to equivalent water depth of 8 g/cm^2^. The absorbed dose rate to water was measured with the PMMA phantom, the spherical water-filled phantom and a commercial solid water phantom. The measured dose rate with the PMMA phantom was 1.2% and 1.8% higher than those measured with the spherical water-filled phantom and the solid water phantom, respectively. These differences can be explained by the scattered photon contribution of PMMA off incoming ^60^Co gamma-rays to the dose rate. The average full width half maximum and penumbra values measured with the PMMA phantom showed reasonable agreement with two calculated values, one at the center of the PMMA phantom (LGP6.93) and other at the center of a water sphere with a radius of 8 cm (LGP8.0) given by Leksell Gamma Plan using the TMR10 algorithm.

**Conclusions:**

A PMMA phantom constructed in this study to measure the absorbed dose rates to water and dose distributions of a GK represents an acceptable and practical alternative for GK dosimetry considering its cost-effectiveness and ease of handling.

## Background

The absorbed dose to water and dose distribution penumbra are essential parameters that need to be accurately determined in radiation therapy because their values are directly applied to build treatment plans. For the measurement of the absorbed dose to water, two protocols are generally used in radiotherapy: TG-51, published by the American Association of Physicists in Medicine (AAPM) in 1999, and TRS-398, published by the International Atomic Energy Agency (IAEA) in 2004 [1, 2]. Both protocols require calibration of the ionization chamber in a water-filled parallelepiped phantom for ^60^Co beam and dosimetric measurement using the calibrated chamber in the user beam with a same or similar phantom. Other conditions required for this measurement, such as the source to surface distance (SSD), field size (FS), reference depth, etc., are well described in the protocols.

However, when we attempted to apply these protocols to the Gamma Knife**®** (GK; Elekta AB, Stockholm, Sweden), we confronted problems in applying the conditions of the protocols. First, the conditions specified for the calibration are no longer valid in a GK. In the GK, approximately 200 gamma-ray beams are focused into the patient positioning system (PPS) calibration center instead of being irradiated along a certain direction. Therefore, it is not possible to define a specific SSD, FS, or other parameters of the standard protocols, and unique conditions must be established. Second, no water phantom has been used in the absorbed dose to water measurement of the GK [3]. This dose is usually measured with phantoms made of two types of plastics, acrylonitrile butadiene styrene (ABS) and solid water (Gamex Model 457, SUN NUCLEAR Co., Melbourne, FL, USA). Although the different radiological characteristics between the plastic phantom and the water phantom must be carefully taken into account, they are neglected in most instances. In order to overcome this factor, the authors developed a spherical water-filled phantom with an equivalent water depth (EWD) of 8 g/cm^2^ and showed that there could be approximately a 2% difference in dose rates measured by the spherical water-filled phantom and two ABS phantoms [4]. Although it is expected that the spherical water-filled phantom provides a more accurate value of the absorbed dose rate to water, there are practical obstacles for using this kind of phantom in ordinary clinical settings due to difficulties in management and risk of damage at the center of the GK during measurement. The solid water phantom (Leksell Gamma Knife Dosimetry Phantom, Elekta, Stockholm, Sweden) represents an alternative because it should have a response to radiation equivalent to water. However, because of its high price, this option is not likely to be generally accepted. Recently, IAEA published a technical reports series no.483 (IAEA TRS-483) [5] and provided a code of practice for dose determination of non-standard fields. IAEA TRS-483 recommended to use plastic phantoms and presented the output correction factors for several combinations of ion chambers and two phantoms from the GK manufacturer. Though it can be accepted as a standard in the clinical sites, it did not provide correction factors for other phantom materials and there are still debates on the exact values of the correction factors such as the study from Mirzakhanian et al. [6].

In this study, we developed a polymethyl metacrylate (PMMA) phantom for GK dosimetry that will be of great use for general GK dosimetry. A calibration factor to convert the measured value to the absorbed dose rate to water was determined by comparing the dose rates of a GK Perfexion measured with the PMMA phantom to those obtained with the spherical water-filled phantom. This factor was also verified by two independent Monte Carlo simulations. For quality assurance of the absolute dose rate measurement and relative dose distribution with the same phantom, two types of inner phantoms were manufactured to accommodate an ionization chamber and radiochromic films.

## Methods

### Construction of the PMMA phantom

A multi-purpose PMMA phantom for measurement of the absorbed dose to water and dose distributions of a GK was constructed. The phantom material was chosen to be PMMA, which was recommended by IAEA as a water substitute and is commonly used in radiation dosimetry. Considering the geometrical distribution of the ^60^Co sources in a GK, the phantom was designed to be cylindrically symmetric, and the radius was intended to be 8.00 g/cm^2^ of equivalent water depth, which was suggested by IAEA/AAPM [5] as the reference depth for measurement of the absorbed dose rate to water in GK dosimetry. The physical radius of the phantom was determined via an electron density comparison between the PMMA and water [7]. The electron density of the PMMA was calculated as follows:

1 $$ {\rho}_{\mathrm{PMMA}}^{\mathrm{el}}={\rho}_{\mathrm{PMMA}}^{\mathrm{mass}}\times \left[\sum \limits_i{f}_i\ {\left(\frac{\mathrm{z}}{A}\right)}_i\right], $$

where ${\rho}_{\mathrm{PMMA}}^{\mathrm{mass}}$ is the bulk density of PMMA, *f*_*i*_ is the fraction by weight of the atom *i*, and ${\left(\frac{\mathrm{z}}{A}\right)}_i$ is the atomic number weight ratio. The radius of the phantom was then obtained from the following equation:

2 $$ {r}_{PMMA}={r}_{EWD}\frac{\rho_{H_2O}^{\mathrm{el}}}{\rho_{PMMA}^{\mathrm{el}}}, $$

where *r*_*PMMA*_ is the physical radius of the PMMA phantom, *r*_*EWD*_ is the equivalent water depth, ${\rho}_{H_2O}^{\mathrm{el}}$ is the electron density of water, and ${\rho}_{PMMA}^{\mathrm{el}}$ is the electron density of PMMA.

**Fig. 1:**
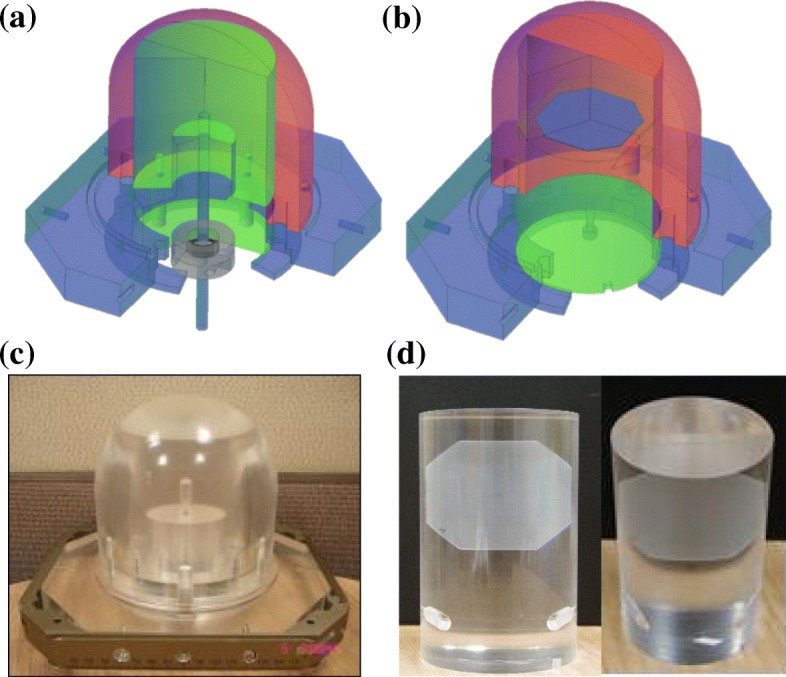
Schematic diagrams and photographs of the PMMA phantom. **a** Design of the phantom with the chamber-hosting inner phantom. **b** Design of the phantom with the xy-plane film-hosting inner phantom. **c** Manufactured phantom combined with the chamber-hosting inner phantom. **d** The xz-plane film-hosting (left) and xy-plane film-hosting inner phantom (right)

The phantom consisted of an outer phantom and exchangeable inner phantoms, as well as one chamber-hosting inner phantom and two film-hosting inner phantoms. One of the film-hosting inner phantoms was designed to set the film in the xy-plane (axial plane) of the Leksell stereotactic coordinate system, and the other was in the xz-plane (sagittal plane). The detailed structure of phantom was drawn using AutoCAD 2D and 3D tools (version 2014, Autodesk Inc.) and the PMMA was machined to a tolerance of 0.002 cm. To minimize deformation of the PMMA due to heat generated by machining, soluble cutting oil was applied continuously during the process. Schematic diagrams of the PMMA phantom and photographs of the outer and inner phantoms are presented in Fig. 1. The radius of the hemispherical portion of the outer phantom was 6.93 cm. The outer phantom was firmly attached to the Leksell G-frame (Elekta AB, Stockholm, Sweden) as shown in Fig. 1c. Each inner phantom was tightly fitted into the outer phantom with the smallest possible air gap to allow the entire phantom to be approximated as homogeneous. There was a directional guide on each phantom to maintain the relative orientation.

### Measurement of absorbed dose to water

**Fig. 2:**
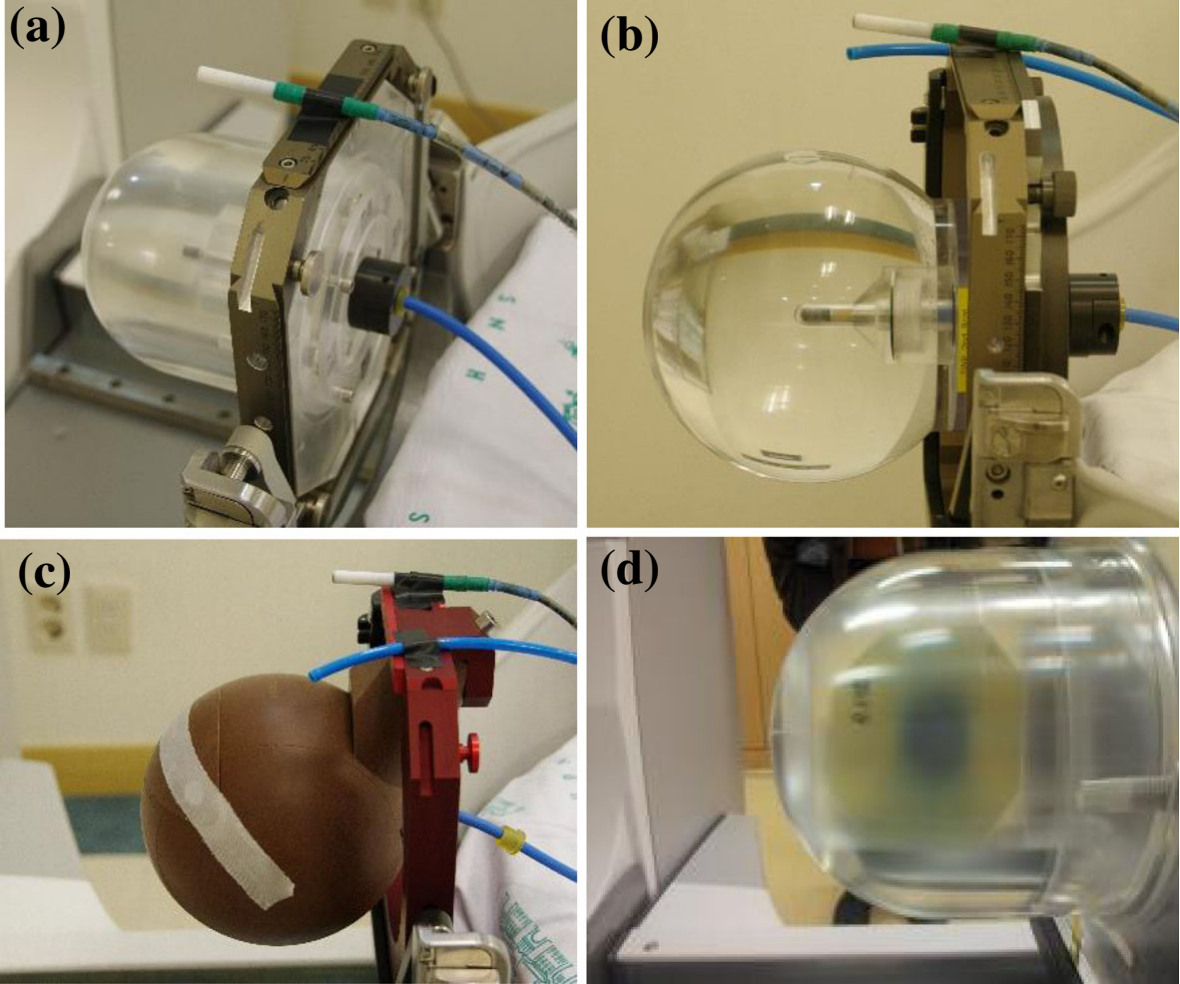
Pictures of the experimental setup for measurement of the absorbed dose to water with different phantoms. **a** The PMMA phantom with a PTW 31010 ionization chamber was mounted to the Leksell Gamma Knife Perfexion for dose rate measurement. **b** The spherical water-filled phantom with a PTW 31010 ionization chamber. **c** A solid water phantom with a PTW 31010 ionization chamber. **d** The PMMA phantom with the xz-plane film-hosting inner phantom. The film shows the dose distribution of the 16-mm collimator in the xz-plane

For the measurement of the absorbed dose rate to water, a PTW TN31010 ionization chamber and a Keithley 6517B electrometer (Keithley Instruments Inc., Cleveland, OH, USA) were used to measure the dose rate of the 16-mm collimator from a Gamma Knife Perfexion. The PTW TN31010, with a nominal sensitive volume of 0.125 cm^3^ and inner radius of 0.275 cm, is recommended for the measurement of dose rate in the GK by the manufacturer. Calibration of the ionization chamber in terms of the absorbed dose to water was carried out at the dosimetry laboratory of the Korea Research Institute of Standards and Science (KRISS), which is a national standard institute in Korea associated with the International Bureau of Weights and Measures (BIPM). A Mensor Model 2105 precision barometer (MENSOR Corp., San Marcos, USA) was used for pressure measurement, and an ASL F250 precision thermometer (Automatic Systems Laboratories, Croydon, UK) was used for temperature measurement. The barometer and thermometer were calibrated annually at the Center for Thermometry and the Center for Mass and Related Quantities, KRISS. Temperature and pressure were measured simultaneously with the ionization current. The absorbed dose rate to water of the 16-mm collimator of a GK Perfexion™ (Elekta AB, Stockholm, Sweden) was measured with the PTW TN31010 chamber inserted into the chamber-hosting inner phantom (Fig. 2a). To estimate the contribution by the scattered photon in the phantom material, the dose rate at the center of a spherical water-filled phantom was also measured (as shown in Fig. 2b) and used as a reference value. The structure and material of the spherical water-filled phantom were described in detail in a previous report [4]. For the comparison, the commercial solid water phantom (Elekta, Sweden) was also used for measurement of the dose rate (Fig. 2c).

### Monte Carlo simulation of the scattering contribution

Seco and Evans [7] reported that the electron-density scaling method considering only Compton scattering can predict primary photon fluences in commonly used plastic materials to within a 0.5% difference from those in water by Monte Carlo simulations with a 1-MeV photon pencil beam with a diameter of 0.1 mm. The remaining discrepancies can be attributed to other scattered photon contributions, and their effects cannot be generally predicted because they depend on various factors such as the phantom geometry, beam shape, and beam energy. In the present study, the effect of the scattered photons in the PMMA phantom was measured by comparing the dose rates measured in the PMMA phantom and the water-filled phantom. To verify the measured value, Monte Carlo simulations were performed with a simplified geometry. The absorbed doses to a water sphere with a diameter of 5.5 mm located at the center of a spherical water phantom with a radius of 8.0 cm and a spherical PMMA phantom with a radius of 6.93 cm were simulated. The diameter of 5.5 mm was chosen to be the same as the diameter of the active volume of the PTW 31010 ionization chamber. The standard chemical composition of PMMA (C_5_O_2_H_8_)_n_ and a mass density of 1.1847 g/cm^3^ were used in the simulation. The geometry of a GK Perfexion was obtained from the vendor. Three independent Monte Carlo simulations were performed. In a simulation with Geant 4 version 10.02 [8], five runs with 1.92 × 10^10^ histories were executed for each phantom, and their averaged values were compared. The PENELOPE low-energy electromagnetic model of Geant4 was used, and the range cut value was 0.1 mm for all kinds of particles. When the dose distributions generated by this simulation were compared with those from Leksell Gamma Plan (LGP) version 11.0 [9], the pixels with absorbed doses larger than or equal to 20% of the maximum dose showed global gamma index pass rates of 99.4% and 99.5% under 1 mm/3% criteria in the axial and sagittal planes, respectively. In another simulation with Geant 4 version 10.02 [8], which was performed by a separate team with independent code generation, a phase space file for the 16-mm collimator along a single direction was built by generating 1.08 **×** 10^10^ photons from the ^60^Co source. The generated phase space file had 3.33 **×** 10^8^ particles. Using the phase space file, six simulation runs with 6.66 **×** 10^8^ histories were performed for each phantom and the averaged values were compared. The physics model of Geant 4 and the range cut value were the same as in the simulation described above. In the third simulation, the user-code penEasy Imaging v. 2010-09-02 [10, 11], which is based on the MC-system, PENetration and Energy Loss of Positrons and Electrons (PENELOPE) 2008 [12], was used. A phase space file (provided by Elekta Instrument AB) describing a 16-mm Perfexion beam was used as the input source. The absorbed dose to a water sphere with a 2.75-mm radius placed in an 8.0-cm spherical water phantom and 6.93-cm PMMA phantom was calculated. The transport parameters used in these MC calculations can be found in Benmakhlouf et al. [13].

### Measurement of the beam profile

The beam profiles using the 16-mm collimator of six GK Perfexion™ units were measured using GafChromic™ MD-V3 films (International Specialty Products, Wayne, NJ, USA). Full width half maximum (FWHM) values and the physical penumbra, which is defined as the distance from the dose level of 80% to the dose level of 20%, were measured [14]. The films were cut into 6 **×** 6-cm^2^ octagonal pieces to be fitted into the film-hosting inner phantom. The films were calibrated by irradiating 5, 10, 15, 20, 25, 30, 35, 40, 45, 50, 55, and 60 Gy at the maximum using the 16-mm collimator. The analysis of the irradiated films followed standard procedures of radiochromic film dosimetry [14]. The films were scanned using an EPSON Expression 10000XL scanner with a transparency unit (Seiko-Epson Co, Nagano, Japan). The scanned images were analyzed with ImageJ and homemade LabVIEW software. More detailed film handling procedures are described in a previous report [15]. In brief, the films were scanned with 300 DPI resolution and the red channel values were converted to optical densities. Absorbed doses were obtained by fitting the calibration data points with a third-order polynomial using the commercial software package, Origin 2015 (OriginLab Corp, Northhampton, MA, USA). One-dimensional dose distributions along the x-axis (right to left), y-axis (posterior to anterior), and z-axis (head to feet) were analyzed and compared with LGP.

## Results

### Construction of the PMMA phantom

**Fig. 3:**
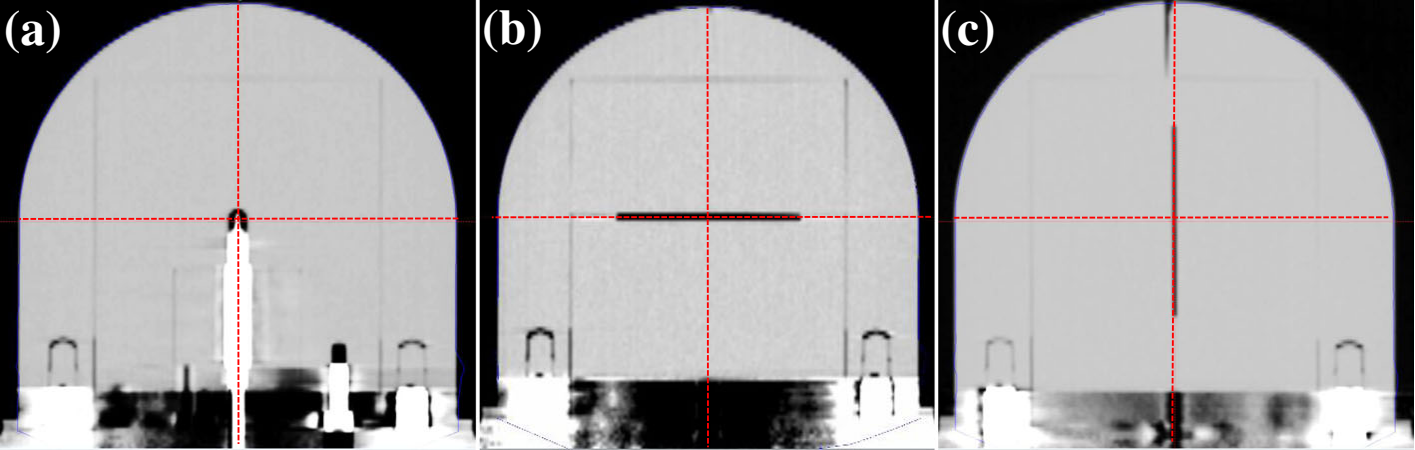
Reconstructed coronal computed tomography images of the PMMA phantom with different inner phantoms. In each figure, the crossing point of the dashed lines is the PPS calibration center of a GK. **a** The effective point of the PTW TN31010 ionization chamber is 0.2 mm off in the x-direction (right-left). **b** The xy-plane film position is well matched with the xy-plane. **c** The xz-plane film position is well matched with the xz-plane

The atomic compositions of the PMMA were analyzed using the element analysis (EA) technique at the Korea Research Institute of Chemical Technology. The bulk density was obtained by measuring the weights of three 0.9981 ± 0.0002 cm^3^ cubic pieces ten times each. The bulk density of the PMMA was 1.185 ± 0.001 g/cm^3^. The atomic compositions of the PMMA and the calculated electron density are shown in Table 1. The unknown material was assumed to be evenly distributed and to have an atomic number weight ratio of 0.5. Its contribution to the resultant electron density was negligible because the actual radius of the phantom was not affected by their existence. The radius of the PMMA phantom was determined to be 6.93 cm for a corresponding equivalent water depth of 8.00 g/cm^2^. The alignment of the center of the active volume of the PTW TN31010 ionization chamber to the PPS calibration center was investigated by computed tomography (CT) images taken using a GE Light Speed Ultra CT (GE Healthcare Korea, Seoul, Korea) (Fig. 3a). The measured coordinate values of the effective point of the PTW 31010 ionization chamber deviated by 0.2 mm along the x-axis only. The deviations of the film-holding inner phantoms were less than or equal to 0.1 mm along a direction perpendicular to the dose distribution measuring plane (Fig. 3b, c).

**Table 1:** Chemical composition and electron density of the PMMA used as the phantom material

Component	fi	Z/A	ρ_e,PMMA_	ρ_e,PMMA_ /ρ_e,water_
H	0.081	0.992162	0.639	1.155
C	0.595	0.499542		
O	0.317	0.500031		
unknown	0.007	0.500		

Legend: *f*_i_ is the fraction by weight of the atom i, Z/A is the atomic number weight mass ratio, *ρ*_e,PMMA_ is the electron density of PMMA and *ρ*_e,PMMA_ /*ρ*_e,water_ is the ratio of the electron densities of PMMA and water

### Measurement of absorbed dose rate to water

The measured absorbed dose rate to water with the 16-mm collimator was 1.666 ± 0.015 Gy/min for the PMMA phantom, 1.647 ± 0.015 Gy/min for the water-filled phantom, and 1.637 ± 0.015 Gy/min for the solid water phantom. The dose rate measured with the PMMA phantom was 1.2% higher than that measured with the spherical water-filled phantom and 1.8% higher than that measured with the solid water phantom.

The experimental factor to convert a measured dose rate in the PMMA phantom to the dose rate in the water-filled phantom was 0.989 ± 0.013. The first Geant4 simulation using no phase space file provided a conversion factor of 0.9883 ± 0.0004, and the second Geant4 simulation using a phase space file showed a conversion factor of 0.9893 ± 0.0006. The PENELOPE-simulated conversion factor was 0.986 ± 0.002. The differences between the simulated conversion factors and the experimental values were 0.1%, 0.0% and 0.3%, respectively.

### Measurement of the beam profile

**Fig. 4:**
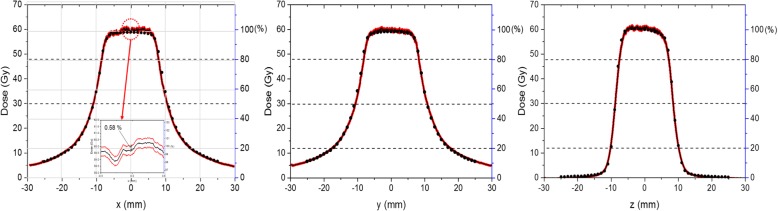
One-dimensional dose distributions using the 16-mm collimator of a Gamma Knife Perfexion. The solid lines indicate measured distributions in the PMMA phantom, and the solid circles indicate results calculated by the GK treatment planning program at the center of a sphere with an 8.0-cm radius. The measured data are expressed a band and its half width is one standard deviation of each point

The optical densities from the dose calibration films were fitted with a third-order polynomial to convert the optical density to the absorbed dose, and the adjusted *R*^2^ value of the fitting curve was 0.9997. Typical one-dimensional beam shapes taken at the center of the PMMA are given in Fig. 4. The calculated beam shapes given by LGP using the TMR10 algorithm are given together [9]. The average values of the FWHM and penumbra measured using the PMMA phantom at six GK Perfexion sites are shown in Table 2.

**Table 2:** FWHM and penumbra values measured in the PMMA phantom and the solid water phantom (SW) and calculated by LGP

	FWHM (mm)				Penumbra (mm)			
Axis	PMMA	LGP6.93^a^	SW	LGP8.0^b^	PMMA	LGP6.93^a^	SW	LGP8.0^b^
x	21.83 ± 0.25	21.68	21.57 ± 0.27	21.75	9.58 ± 0.22	9.04	8.76 ± 0.19	9.03
y	22.10 ± 0.32	21.68	21.56 ± 0.27	21.75	9.86 ± 0.18	9.04	8.96 ± 0.23	9.03
z	17.46 ± 0.11	17.39	17.41 ± 0.17	17.44	2.72 ± 0.10	3.09	2.65 ± 0.14	2.55

Legend: ^a^Calculated by Leksell Gamma Plan at the center of the PMMA phantom (LGP6.93)

^b^Calculated by Leksell Gamma Plan and at the center of a sphere with a radius of 8.0 cm (LGP8.0)

For comparison, measured values using the solid water phantom at a single GK Perfexion site are also given. Two calculated results, one at the center of the PMMA phantom (LGP 6.93) and the other at the center of a water sphere with a radius of 8.0 cm (LGP 8.0), were obtained in the dose distributions given by LGP using the TMR10 algorithm. The TMR10 algorithm took into account only the geometry of the phantoms and neglected inhomogeneous electron densities between materials.

## Discussion

### Measurement of the absorbed dose rate to water

The difference between the absorbed dose rate to water measured with the PMMA phantom and those with the water-filled spherical phantom and the solid water phantom can be explained by the radius of the PMMA phantom having been determined based on elemental analysis of the PMMA, bulk density measurement and electron density calculation. However, the scattered effect of the PMMA body off the incoming ^60^Co beam could cause additional contribution to the measurement of dose rate at the center of the PMMA phantom, whereas the photon beam scattered from the thin (5 mm) spherical PMMA shell of the water-filled phantom was attenuated mostly by the water before it arrived to the chamber at the center of the water-filled phantom. Therefore, the 1.2% difference seemed to be mainly due to the scattered photon contribution. Another possible reason for this difference may be the geometrical effect between the PMMA phantom and the water-filled phantom, although the hemispherical geometry of the PMMA phantom was nearly identical to the spherical shape of the water-filled phantom as long as the effective area of the GK is concerned, and its contribution to the dose rate should be negligible.

The comparison of the measured dose rate between the solid water phantom and the spherical water-filled phantom showed a difference of 0.6%, and this could also be due to the scattered photon contribution of the solid water material to the dose rate. These results are consistent with the recently reported round-robin dose rate measurement in which the dose rate measured with a PTW 31010 ionization chamber in a hemispherical liquid water phantom was 0.4% larger than the dose rate measured in the Elekta solid water phantom under the TG-51 protocol [16].

### Measurement of the beam profile

The FWHM values measured in the PMMA phantom were closer to the calculated values at the center of the 8.0-cm sphere than the values measured in the solid water phantom. However, all the FWHM values were within a range with variations less than 0.4 mm. Penumbras showed larger variations from the calculated values. In general, the PMMA showed wider penumbra than those from LGP8.0, while the values from the solid water phantom were narrower. This difference was due to the wider width of the 20% dose line in the PMMA phantom, which can be explained by the greater scattering contribution in the PMMA phantom compared to that in phantoms composed of other materials.

### Uncertainty analysis

A detailed uncertainty budget based on the relative standard uncertainties for the measurements of the absorbed dose rate to water and the beam profile are tabulated in Tables 3 and 4. The contributing uncertainty components were classified as either statistical (type A) or systematic (type B) uncertainties, and their values are listed [17]. The main features of the uncertainty components are described as follows.

**Table 3:** The uncertainty components for measurement of the absorbed dose rate to water

Uncertainty component	Type A (%)	Type B (%)
Calibration factor of the ionization chamber, ${N}_{D_w,{Q}_0}^c$		0.50
Ionization current measurement	0.01	0.02
Temperature and pressure measurement		0.056
Scattered photon contribution	0.01	1.28
Displacement of the ionization chamber	0.01	0.15
Alignment of the PMMA phantom with the PPS calibration center		0.20
Long-term stability of the ionization chamber		0.12
Standard relative combined uncertainty	1.40	

Legend: A PTW TN31010 ionization chamber was used for measurements of the FWHM and penumbra at the patient positioning system (PPS) calibration center

**Table 4:** The uncertainty components for measurements of the FWHM and penumbra

Uncertainty component	Type A (%)	Type B (%)
Optical density measurement	0.10	0.02
Intensity resolution		0.002
Uniformity of the radiochromic film		0.50
Temperature and relative humidity measurements		0.056
Alignment of the PMMA phantom with the PPS calibration center		0.20
Positioning of the radiochromic film inside the phantom		0.16
Calibration curve fitting		1.33
Measurements of FWHM and penumbra	0.02	0.01
Standard relative combined uncertainty	1.45	

Legend: GafChromic™ MD-V3 films were used for the measurement of absorbed dose rate to water at the patient positioning system (PPS) calibration center

The uncertainty for the calibration factor of the chamber may be provided in the calibration certificate. The uncertainty for the displacement of the ionization chamber can be measured by moving the reference phantom in 0.1-cm steps. Although the change in the dose rate in the x- and y-axes was less than 0.03%, the change along the z-axis was 0.15%. The uncertainty of the alignment of the PMMA phantom with the PPS calibration center of the GK was determined by the difference of the coordinates between the phantom center at (99.8, 100, 100) and the PPS calibration center at (100, 100, 100).

The uncertainty of calibration curve fitting represents the overall uncertainty introduced during the fitting procedure performed to obtain the calibration curve.

Using the PMMA phantom proposed in this study for GK dosimetry may be a good choice because it provides a solution to the problem for how the dose rate should be measured in a water phantom. However, the other conditions required by the standard protocols, such as a single directional beam, source to surface distance of 100 cm, and measurement depth of 5 g/cm^2^, cannot be achieved yet. To do so would require an additional factor to correct these differences by applying a dose correction factor measured in a standard library at a clinical site. Or, the generalized beam quality correction factor suggested by Alfonso et al. [18] could be obtained by Monte Carlo simulations considering the conditions of field size, geometry, phantom material, and beam quality. However, there are no published reports on this generalized correction factor for the GK, except for a technical report and a study on relative output correction factors for different size collimators [13, 19].

## Conclusions

A PMMA phantom was constructed for the measurement of the absorbed dose rates to water and the dose distribution of a GK. The phantom was characterized with the composition of the hemispherical outer phantom fixed firmly to the Leksell Gamma frame and the exchangeable inner phantoms. The radius of the PMMA phantom corresponding to an equivalent water depth of 8 g/cm^2^ was determined from the electron density calculation, but a factor of 0.989 was necessary to convert the measured value to a dose rate absorbed to water. The FWHM and penumbra values measured using the phantom showed reasonable agreement to the calculated values, although more sophisticated handling of the scattered photon contribution seemed to be necessary to explain the wider penumbra in the measured beam shapes. The PMMA phantom in this study represents an acceptable and practical alternative for GK dosimetry considering its cost-effectiveness and ease of handling. For reference, the cost of manufacturing the phantom including the PMMA price and phantom design was forty thousand dollars, which is economical compared with that of purchasing a commercial phantom. The phantom is ready to be commercialized upon request.

## Abbreviations

(AAPM): American Association of Physicists in Medicine (AAPM)
ABS: Acrylonitrile butadiene styrene
BIPM: International Bureau of Weights and Measures
CT: Computed tomography
EWD: Equivalent water depth
FS: Field size
FWHM: Full width half maximum
GK: Gamma knife
IAEA: International Atomic Energy Agency
KRISS: Korea Research Institute of Standards and Science
PMMA: Polymethyl metacrylate
PPS: Patient positioning system
SSD: Source to surface distance

## References

[1] Almond PR, Biggs PJ, Coursey BM (1999). AAPM’s TG-51 protocol for clinical reference dosimetry of high-energy photon and electron beams. Med Phys.

[2] Andreo P, Burns DT, Hohfeld K (2004). Absorbed dose determination in external beam radiotherapy: an international code of practice for dosimetry based on standards of absorbed dose to water. Technical report series no. 398 (V.11b).

[3] Maitz AH, Wu A, Lunsford LD (1995). Quality assurance for gamma knife stereotactic radiosurgery. Int J Radiat Oncol Biol Phys.

[4] Chung HT, Park Y, Hyun S, al e (2011). Determination of the absorbed dose to water for the 18-mm helmet of a gamma knife. Int J Radiat Oncol Biol Phys.

[5] Andreo P, Burns DT, Hohfeld K (2017). Dosimetry of small static fields used in external beam radiotherapy: an international code of practice for reference and relative dose determination. Technical report series no. 483.

[6] Mirzakhanian L, Benmarkhlouf H, Tessler F (2018). Determination of k_Qmsr, Qo_ factors for ion chambers used in the calibration of Leksell gamma knife Perfextion model using EGSnrc and PENELOPE Monte Carlo codes. Med Phys.

[7] Seco J, Evans PM (2006). Assessing the effect of electron density in photon dose calculations. Med Phys.

[8] Allison J, Amako K, Apostolakis J (2016). Recent developments in GEANT4. Nucl Instrum Methods Phys Res A.

[9] Xu AY, Bhatnagar J, Bednarz G (2014). Dose difference between the three dose calculation algorithms in Leksell gamma plan. J Appl Clin Med Phys.

[10] Badal-Soler A. Development of advanced geometric models and acceleration technique for Monte Carlo simulation in Medical Physics. Ph.D. Thesis. Barcelona: Universitat Politècnica de Catalunya; 2008.

[11] Sempau J, Badal A, Brualla L (2011). A PENELOPE-based system for the automated Monte Carlo simulation of clinacs and voxelized geometries application to far-from-axis fields. Med Phys.

[12] Salvat F, Fernandez JM, Sempau J (2009). PENELOPE-2008, A code system for Monte Carlo simulation of electron and photon transport.

[13] Benmakhlouf H, Johansson J, Paddick I (2015). Monte Carlo calculated and experimentally determined output correction factors for small field detectors in Leksell gamma knife Perfexion beams. Phys Med Biol.

[14] Niroomand-Rad A, Blackwell CR, Coursey BM (1998). Radiochromic film dosimetry: recommendations of AAPM radiation therapy committee task group 55. American Association of Physicists in Medicine. Med Phys.

[15] Chung JP, Oh SW, Seong YM (2016). An effective calibration technique for radiochromic films using a single-shot dose distribution in gamma knife. Physica Medica.

[16] Drzymala RE, Alvarez PE, Bednarz G (2015). A round-robin gamma stereotactic radiosurgery dosimetry inter-institution comparison of calibration protocols. Med Phys.

[17] EAL Task Force. Public Reference, Expression of the uncertainty of measurement in calibration, European co-operation for accreditation, EA-4/02; 1999.

[18] Alfonso R, Andreo P, Capote R (2008). A new formalism for reference dosimetry of small and nonstandard fields. Med Phys.

[19] Johansson J and Gorka B. Application of a new formalism for absolute dosimetry of the Leksell Gamma Knife. Stockholm: Elekta Instrument AB; 2012. SE-10393, Reference No. SSM 2010/2201 and project No. 4017003–06.

